# High-Throughput Identification of Antibacterials Against *Pseudomonas aeruginosa*

**DOI:** 10.3389/fmicb.2020.591426

**Published:** 2020-12-09

**Authors:** Shijia Li, Pengfei She, Linying Zhou, Xianghai Zeng, Lanlan Xu, Yaqian Liu, Lihua Chen, Yong Wu

**Affiliations:** Department of Laboratory Medicine, The Third Xiangya Hospital, Central South University, Changsha, China

**Keywords:** *Pseudomonas aeruginosa*, demeclocycline hydrochloride, SAAP-148, high-throughput screening, drug combination

## Abstract

Antibiotic resistance is a growing public health concern, though the constant development of new antibiotics. The combination of high-throughput screening and drug repurposing is an effective way to develop new therapeutic uses of drugs. In this study, we screened a drug library consisting of 1,573 drugs already approved by the Food and Drug Administration and 903 drugs from the natural product library, to identify antimicrobials against *Pseudomonas aeruginosa*. A high-throughput screening assay based on microtiter plate was used to screen 39 drugs that inhibit the planktonic or biofilm formation of *P. aeruginosa* while most of them are antibiotics. The antimicrobial activities of these drugs were evaluated by phenotypic analysis. Further studies showed the combined therapy of tetracycline antibiotics demeclocycline hydrochloride (DMCT) and the novel antimicrobial peptide SAAP-148 has an effective synergistic antibacterial effect on *P. aeruginosa* PAO1 and *P. aeruginosa* ATCC27853. Moreover, the time-kill curve assay and murine model of cutaneous abscesses further confirmed the synergistic effect. In addition, the combination of DMCT and SAAP-148 has the potential to combat clinically isolated multidrug-resistant (MDR) *P. aeruginosa* strains. Our results clearly indicate that DMCT and SAAP-148 combined therapy could be an effective method to combat MDR *P. aeruginosa*-related infections.

## Introduction

*Pseudomonas aeruginosa* is a Gram-negative opportunistic pathogen usually widespread in clinical settings (Rahme et al., 1995), causing life-threatening infections in diverse patient populations (Djapgne et al., 2018). *P. aeruginosa* can grow as single cell in planktonic form or in a physiologically different biofilm form (She et al., 2019). Bacterial biofilm is a bacterial community that contains a large number of densely packed cells (Prindle et al., 2015), and it is difficult to remove with antibacterial agents due to its antibiotic or biocide resistance relative to planktonic cells (Mah and O’Toole, 2001; Chu et al., 2016). As *P. aeruginosa* is prone to intrinsic and acquired antibiotic resistance that’s why it has become one of the most difficult opportunistic pathogens to treat, and can cause many serious infections in immunocompromised and hospitalized patients (Pendleton et al., 2013; Sousa and Pereira, 2014). Moreover, the formation of biofilm makes *P. aeruginosa* infection chronic.

Antibiotics can be considered as the greatest medical intervention in human history (Pletzer et al., 2018). Although the current global health assessment shows that the number of infections caused by bacteria resistant to many antibiotics has increased significantly (Almaaytah et al., 2019). Drug discovery is a key process to discover compounds and molecules that may develop into clinical therapeutic drugs. However, this development process is expensive and full of risks of failure (Trombetta et al., 2018). Therefore, there is an urgent need to replace antibiotics or change current treatment methods to prevent further resistance and/or improve the effects of existing antibiotics (Hayes et al., 2019). Repurposing drugs that have been applied to humans is a promising method that can reduce the cost and time usually associated with traditional drug discovery strategies, and is more likely to produce bioavailable and safe compounds, which can be more convenient and faster to enter clinical trials (Ashburn and Thor, 2004; Mullard, 2012).

In addition to drug repurposing, one of the drugs that have been intensively studied for the successful development of antibiotics in the past decade is antimicrobial peptides (Mishra et al., 2017), which has received increasing attention as potential novel antibiotics (Fosgerau and Hoffmann, 2015). At present, more and more synthetic antibacterial peptides are reported. One of the presumed advantages of AMPs is that the evolution of bacterial resistance is much slower than that of traditional antibiotics, which is a very desirable characteristic (Yu et al., 2018). The antimicrobial peptide SAAP-148 is an LL-37-inspired peptide developed by de Breij’s team (de Breij et al., 2018). They confirmed that SAAP-148 has a wide range of antibacterial activity against Gram-positive and Gram-negative bacteria, including *P. aeruginosa*, so it is an ideal drug to combat fighting difficult-to-treat infections. Since the use of antibiotics can easily lead to bacterial resistance, the combined use of antimicrobials is an attractive option with many advantages, including potential synergistic effects. Therefore, the combined use of antimicrobials can improve the efficacy of either monotherapy (Brooks and Brooks, 2014). The combination therapy can also potentially reduce the concentration required to treat bacterial infection, hereby reducing future antibiotic resistance. In the absence of evidence-based treatment options, combinations are increasingly employed to enhance the antibacterial effects of available drugs against multidrug-resistant strains (Tängdén, 2014).

In this work, we used a high-throughput screening assay to screen FDA-approved products to identify drugs with novel antimicrobial activity against *P. aeruginosa* planktonic or biofilms. After verifying the antibacterial effect through phenotypic experiments, we chose the tetracycline antibiotic DMCT for further analysis. We evaluated the antibacterial activity of DMCT used in combination with SAAP-148, and tested against *P. aeruginosa* PAO1, ATCC27853 and current clinical bacterial strains through checkerboard assay and time-kill assay. Finally, the effectiveness of the combined therapy was verified by the *in vivo* experiment of the murine cutaneous abscess model.

## Materials and Methods

### Bacterial Isolates, Cultural Conditions, and Reagents

The strains used are laboratory strain PAO1 (ATCC15692), a widely used and well-characterized wound isolate, and *P. aeruginosa* ATCC27853 (Holloway, 1955; Schmidt et al., 1996). The clinical isolates used in this study were obtained from the sputum or pus of inpatients at the Third Xiangya Hospital of Central South University (Changsha, Hunan, China). *P. aeruginosa* strains were grown in Luria broth (LB) (Solarbio, Shanghai, China) at 37°C with constant shaking (180 rpm), and the peptides were synthesized by GL Biochem (Shanghai, China).

### High-Throughput Screening

A library of 2,476 FDA approved drugs was purchased from MedChem Express (Monmouth Junction, NJ, United States), and preliminary analysis was performed in a 96-well microtiter plate (Corning costar, Cambridge, MA, United States). *P. aeruginosa* PAO1 strain was used for high-throughput screening. Briefly, overnight cultures of *P. aeruginosa* PAO1 were suspended in MH broth to 1 × 10^6^ CFU/ml. Hundred microliter of bacterial suspension was added to 96 μl of MH broth, and 4 μl of screening drugs from the drug library was added to wells of a 96-well microtiter plate, achieving a concentration of 100 μM. The plate was then incubated at 37°C for 24 h. After treatment, we used the turbidimeter to determine the survival rate of planktonic cells at an optical density (OD) of 630 nm (Campbell, 2011; Sun et al., 2016) and classified the drugs that reduce the turbidity of culture by more than 50% as compared with the untreated control as the plate was gently washed with 1 × PBS after the culture was removed. The inhibition rate of biofilm formation was determined by measuring at 570 nm of 0.5% (w/V) crystal violet dissolved in ethanol. Experimental assays were performed twice. The second screening reduced the drug concentration to 30 μM. The screening procedure was the same as the initial screen. Experimental assays were performed in triplicate.

### Antimicrobial Susceptibility Assay

According to the Clinical and Laboratory Standards Institute (CLSI) guidelines (Institute, 2006), the *in vitro* antibacterial activity of selected drugs was evaluated by microdilution broth sensitivity test (Institute, 2006). The MIC values of the selected drug were determined by the wells with the lowest concentration and no visible bacterial growth. To determine MBC, bacterial cultures from the wells were mixed gently and streaked on blood agar plates, and then the plates were incubated at 37°C for 24 h. The MBC value was defined as the minimum drug concentration without bacterial colony growth on the plate after 24 h of incubation (Huang et al., 2012). The assay was conducted in triplicate.

### Frequency of Resistance

The spontaneous resistance frequency was performed based on the modified method reported by Peter A. Smith with slight adaptations (Smith et al., 2018). Five independent colonies from PAO1 were scraped off and established an overnight culture. The cultures were concentrated to 1 × 10^9^ CFU/ml by centrifuging and resuspending in 100 μl. The concentrated bacterial cell suspensions were then spread on MH agar containing drugs at 4–8-times the MIC. The MH plates were prepared from Mueller Hinton II cation adjusted broth (BBLTM 212322) and SeaKem LE Agarose (17 g/L; Lonza) as per manufactures instructions. The resistance frequency was calculated by dividing the number of colonies formed after a 48 h incubation at 37°C by the initial viable cell count. Testing was performed in triplicate.

### Biofilm Determination

For *P. aeruginosa* determination, bacterial cultures were grown overnight and diluted 1:100 in MH broth (Included 1% glucose). 200 μl of the diluted cultures were added to each well of the microtiter plate and incubated at 37°C for 24 h. After the incubation, the cultures in the wells were aspirated and rinsed, 100 μl of MH broth and 100 μl of the selected drug were added to each well, and incubated for 37°C for 24 h. Then washed away the liquid in the well and determined the remaining biofilm by XTT assay. In short, XTT and phenazine methyl sulfate were diluted to a concentration of 0.2 and 0.02 mg/ml with 1 × PBS, respectively, and then mixed (MACKLIN, Shanghai, China). Then 200 μl of the mixture was then added to the wells, and measured the contents at OD490 nm after incubating for 3 h in the dark at 37°C (Nesse et al., 2015). MBEC50/MBEC70 was defined as the lowest concentration for a specific drug to inhibit 50%/70% growth of biofilms compared to the untreated group (Gomes et al., 2009).

### Checkerboard Assay

The efficacy of individual drug and the combined therapy with SAAP-148 were evaluated by using the microdilution checkerboard assay (Odds, 2003). According to the MIC, the maximum concentration of the drug is twice the MIC value. Overnight cultures were diluted in MH broth to reach a final concentration of approximately 5 × 10^5^ CFU/ml. The fractional inhibitory concentration index (FICI) is measured by dividing the combined MIC by the MIC of a single antibacterial agent. FICI ≤ 0.5 designated indicates synergy; 0.5 < FICI < 1.0 designated partial synergy; FICI = 1.0 designated additivity; 1.0 < FICI < 4.0 designated indifference; and FICI ≥ 4.0 designated antagonism (Pachón-Ibáñez et al., 2011).

### Time-Kill Assay

To confirm the synergistic effects of DMCT and SAAP-148 on PAO1 and ATCC27853, a time-kill assay was carried out. The overnight bacterial cells were diluted into 10 ml aliquots in a burette with a density of 1 × 10^6^ CFU/ml. 10 ml of MH broth containing 1/2×, 1× or 2 × MIC drugs was inoculated individually or in combination with a final concentration of 1 × 10^5^ CFU/ml. The negative control was contained with equal amount of saline. During the incubation period, 100 μl of each sample was taken at 0, 2, 4, 8, 12, and 24 h of incubation at 37°C. Each sample was serially diluted 10-fold in aliquots and plated on blood agar, incubated overnight at 37°C for colony counts.

### Hemolysis Assay

The hRBCs were collected from the Department of Clinical Laboratory of the Third Xiangya Hospital. 2 ml hRBCs were washed 3 times using 1 × PBS followed by centrifugation for 5 min at 1,000 rpm at 4°C. The hRBCs were then added to SAAP-148 containing 1 × PBS (1.5625 μM to 200 μM) for a final hRBCs concentration of 5% (vol/vol). After incubation for 1 h at 37°C, the samples were centrifuged at 1,000 rpm for 5 min, then 100 μl of the supernatant was added to the 96-well plate and the absorbance was measured at 450 nm. The hRBC samples with 0.1% Triton X-100 or 1% DMSO were used as positive and negative controls, respectively. Three replicates were evaluated under this condition.

### Cutaneous Mouse Infection Model

The mice used in this study were purchased from Hunan Slake Jingda Experimental Animal, Co., Ltd. (Hunan, China). All mice were 6-week-old, female CD-1, weighing about 25 ± 3 g during the experiment. The infection model has been slightly modified, as described in previous reports (Pletzer et al., 2018). The bacterial cultures used in the infection model were washed twice with 1 × PBS and then suspended in saline. To produce a repeatable abscess, 100 μl bacterial suspension was injected into the right side of the dorsum to achieve a final concentration of about 1 × 10^9^ CFU/mice. The treatment was delivered directly into the subcutaneous space to the infected area (100 μl) at 1 h post-infection. The development of infection was monitored daily. Skin abscesses (including all empyema) were excised and regimented homogenized in 1 × PBS by an automatic tissue homogenizer (Servicebio KZ-II, Wuhan, China). Bacterial count was quantified by serial dilution. Hematoxylin and eosin (H&E) staining is used for histopathological analysis of skin abscesses. The experiment was performed at least three times, with 2–4 animals in each group.

### Statistical Analysis

Statistical analysis was performed using GraphPad Prism 6.0. Statistical significance was analyzed by the ANOVA test. *P* < 0.05 was considered a statistically significant difference.

## Results

### Identification of Screening Drugs That Are Bactericidal Against *P. aeruginosa*

The high-throughput *in vitro* screening identified FDA-approved drugs that effectively inhibit *P. aeruginosa* PAO1 planktonic or biofilm cell. We identified the growth inhibitors of PAO1 through 96-well microtiter plate-based high-throughput screening of 2,476 compounds belonging to the compound library. The initial screening drug concentration was 100 μM, and we identified 67 compounds that inhibit PAO1 plankton or biofilm by > 50%, as determined by turbidometry (Supplementary Table S1). The second screening reduced the concentration of 67 compounds to 30 μM, and a total of 39 drugs with an inhibition rate of ≥ 50% were screened out (Table 1). Not surprisingly, most of the compounds are known as antibiotics.

**TABLE 1:** Drugs with activity against *P. aeruginosa* PAO1 planktonic and biofilm.

**Drug**	**Class**	**% planktonic inhibition at 30 μ M**	**% biofilm inhibition at 30 μ M**	**MIC (μ M)**	**MBC (μ M)**
Tosufloxacin	Fluoroquinolone	100	100	3.125	6.25
Sitafloxacin	Fluoroquinolone	100	98.5	1.5625	3.125
Tobramycin	Aminoglycoside	99.5	96.2	0.78125	3.125
Sisomicin	Aminoglycoside	100	97.7	0.78125	6.25
Amikacin	Aminoglycoside	100	99.6	1.5625	6.25
Azlocillin	Penicillin	100	93	6.25	12.5
Gemifloxacin	Fluoroquinolone	100	97.6	3.125	3.125
Netilmicin	Aminoglycoside	100	96.2	3.125	6.25
Cefozopran	Cephalosporin	100	91.7	0.78125	1.5625
Cefepime	Cephalosporin	100	93.3	1.5625	3.125
Prulifloxacin	Fluoroquinolone	100	97	3.125	6.25
Doripenem	Carbapenem	100	96.3	1.5625	6.25
Colistin	Polymyxin	100	99.8	1.5625	12.5
Ceftazidime	Cephalosporin	100	97	1.5625	3.125
Danofloxacin	Fluoroquinolone	98.2	94.3	6.25	12.5
Piperacillin	Penicillin	100	98.6	1.5625	6.25
Mezlocillin	Mezlocillin	98.4	90.8	25	50
Ceftriaxone	Cephalosporin	100	97	6.25	25
Cefmenoxime	Cephalosporin	100	97.8	3.125	6.25
Lomefloxacin	Fluoroquinolone	100	100	6.25	12.5
Pazufloxacin	Fluoroquinolone	99.2	94.8	6.25	12.5
Aztreonam	Monolactam	100	93.4	6.25	12.5
Garenoxacin	Quinolone	100	95.6	6.25	25
Gatifloxacin	Quinolone	100	98.9	6.25	25
Sparfloxacin	Quinolone	99.1	92.1	6.25	25
Demeclocycline	Tetracycline	100	99.6	12.5	> 50
Rifaximin	Ansamycin	100	99.5	12.5	> 50
Streptomycin	Aminoglycoside	100	97.5	3.125	6.25
Paromomycin	Aminoglycoside	100	94.3	12.5	25
Levofloxacin	Quinolone	100	97.6	12.5	12.5
Pefloxacin	Quinolone	100	93.4	12.5	25
Besifloxacin	Fluoroquinolone	100	92	12.5	25
Enoxacin	Fluoroquinolone	100	91.1	12.5	50
Bekanamycin	Aminoglycoside	100	99.4	12.5	50
Erythromycin	Monobactam	98.8	88.4	50	50
Telithromycin	Macrolide	100	< 80	50	> 50
Azithromycin	Macrolide	100	95	50	> 50
Nedaplatin	Cancer	81	< 80	50	> 50
Chlorhexidine	Fungicide	100	98.8	12.5	25

### Evaluation of Antibacterial Effects of Selected Drugs on *P. aeruginosa*

**FIGURE 1:**
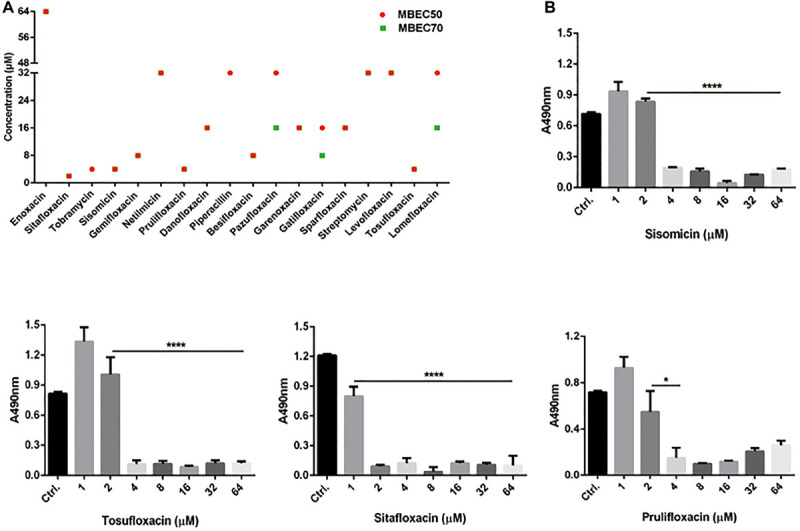
Minimum biofilm eradication concentrations of drugs against *P. aeruginosa* PAO1 Biofilms. **(A)** Biofilm eradication by selected drugs detected by XTT assay. Biofilms cultured for 24 h were treated with selected drugs at the specified concentrations. After incubation, removed planktonic cells and stained biofilms with XTT/PMS solution. The red circle and the green square represent the MBEC50 and MBEC70 of 18 drugs, respectively. **(B)** The effect of representative drugs on the biofilm eradication of PAO1, data representing three independent experiments performed in duplicate. *P < 0.05; ****P < 0.0001.

To further understand the effect of screening drugs on *P. aeruginosa*, a series of phenotypic experiments were conducted. The MIC and MBC assays were determined (Table 1). Since most of the selected drugs inhibited the formation of the PAO1 biofilms, we next evaluate whether they have the ability to eradicate pre-formed biofilms. Quantitative analysis using XTT assay revealed that 18 of the 39 drugs significantly attenuated metabolic activity due to biofilm disruption at the concentration ≤ 64 μM (Figure 1).

The mutation is a common mechanism by which bacteria develop resistance to antibiotics (Fajardo-Cavazos and Nicholson, 2016). The spontaneous resistance frequencies of selected drugs were measured at 4 × and 8 × MIC, to investigate the development of resistant mutants when PAO1 is treated with each drug (Table 2). These data may have a certain guiding role in the selection of medication.

**TABLE 2:** Spontaneous resistance frequencies of *P. aeruginosa* PAO1.

**Drug**	**Spontaneous resistance frequency (SD)**
	**4 × MIC**
Amikacin	4.35 × 10^–6^ (± 1.67 × 10^−6^)
Netilmicin	1.08 × 10^−6^ (± 0.17 × 10^−6^)
Sisomicin	2.57 × 10^−7^ (± 0.9 × 10^−7^)
Streptomycin	6.49 × 10^−6^ (± 1.33 × 10^−6^)
Tobramycin	1.87 × 10^−6^ (± 0.62 × 10^−6^)
Tosufloxacin	0.87 × 10^−7^ (± 0.31 × 10^−7^)
Gemifloxacin	6.73 × 10^−7^ (± 1.4 × 10^−7^)
Chlorhexidine	1.33 × 10^−8^ (± 0.58 × 10^−8^)
Lomefloxacin	2.28 × 10^−6^ (± 0.76 × 10^−6^)
Gatifloxacin	1.23 × 10^−7^ (± 0.38 × 10^−7^)
Telithromycin	2.94 × 10^−6^ (± 0.9 × 10^−6^)
Bekanamycin	9.44 × 10^−6^ (± 7.56 × 10^−6^)
Rifaximin	0.87 × 10^−6^ (± 0.16 × 10^−6^)
Aztreonam	0.33 × 10^−8^ (± 0.58 × 10^−8^)
Pefloxacin	1.37 × 10^−7^ (± 1.06 × 10^−7^)
Garenoxacin	3 × 10^−8^ (± 3.61 × 10^−8^)
Enoxacin	2.27 × 10^−7^ (± 0.75 × 10^−7^)
Sparfloxacin	1.27 × 10^−7^ (± 0.15 × 10^−7^)
	**8 × MIC**
Ceftazidime	1.6 × 10^−6^ (± 0.02 × 10^−6^)
Paromomycin	2.41 × 10^−6^ (± 0.37 × 10^−6^)
Cefmenoxime	5.17 × 10^−7^ (± 3.15 × 10^−7^)
Danofloxacin	4.13 × 10^−7^ (± 0.4 × 10^−7^)
Mezlocillin	2.11 × 10^−5^ (± 0.12 × 10^−5^)
	**4 × MIC**
Sitafloxacin	/
Colistin	/
Prulifloxacin	/
Pazufloxacin	/
Besifloxacin	/
Levofloxacin	/

*/means no colony growth on the plate at 4 × MIC after 48 h incubation at 37°C, drugs that are not listed indicate that the colony is covered with the plate at 4 × MIC after 48 h incubation at 37°C.*

### Synergistic Antibacterial Effect of Drug Combination Against *P. aeruginosa*

The emergence of antibiotic resistance has become a serious public health concern worldwide. Finding novel antimicrobial agents and therapies based on synergistic combinations are essential to combat drug-resistant bacteria. AMPs are one of the most promising antibiotic agents. Therefore, several antimicrobial peptides were selected to determine the antimicrobial activity against PAO1. According to the antibacterial effect of AMPS, SAAP-148 inhibited the growth of PAO1 at low concentrations among selected AMPs. In addition, the cytotoxicity of SAAP-148 was tested by measuring the ability to cause hRBC lysis. We found that SAAP-148 has little hemolytic activity at high concentrations (200 μM) (Supplementary Figure S1). We finally regarded SAAP-148 as an optimized antimicrobial peptide for further detailed research (Supplementary Table S2).

**FIGURE 2:**
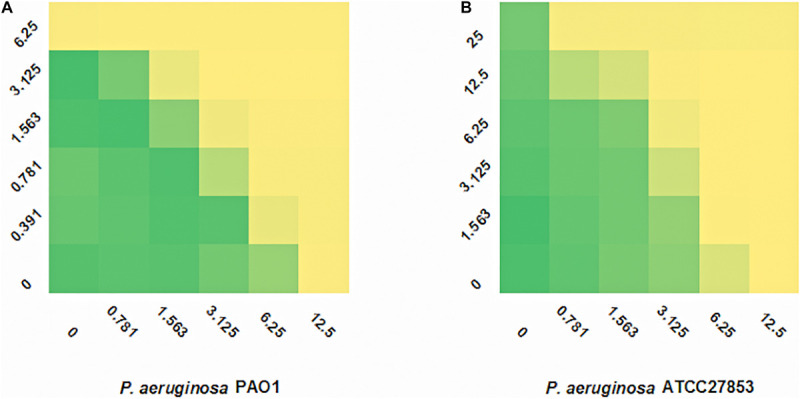
FIC indexes of DMCT/SAAP-148 combination. Fractional inhibitory concentration (FIC) testing of DMCT and SAAP-148 alone and combination against PAO1 **(A)** and ATCC27853 **(B)**.

SAAP-148 is a novel antibacterial peptide with broad-spectrum antibacterial effect. The microdilution checkerboard assay was used to evaluate the synergistic effects of the selected drugs and SAAP-148 (Table 3). The combination of SAAP-148 and DMCT has a significant synergistic effect on PAO1, with a FICI of 0.5 (Figure 2). In addition, we measured another laboratory strain *P. aeruginosa* ATCC27853 by checkerboard assay, synergistic effects or addictive effects can be seen among *P. aeruginosa* ATCC27853 and MDR clinical strains when using the combination of SAAP-148 and DMCT (Supplementary Table S3 and Figure 2).

**TABLE 3:** FICs of selected drugs and SAAP-148 alone or in combination against PAO1.

**Drug**	**MIC (μ M)**		**MIC_In combination_/MIC_singly_**	**FICI**	**Outcome**
	**Singly**	**Combination**			
Demeclocycline	12.5	3.125	0.25	0.5	Synergy
SAAP-148	6.25	1.5625	0.25		
Azlocillin	6.25	3.125	0.5	0.75	Partial Synergy
SAAP-148	6.25	1.5625	0.25		
Mezlocillin	25	6.25	0.25	0.75	Partial Synergy
SAAP-148	6.25	3.125	0.5		
Ceftriaxone	6.25	3.125	0.5	0.75	Partial Synergy
SAAP-148	6.25	1.5625	0.25		
Cefepime	1.5625	0.78125	0.5	0.75	Partial Synergy
SAAP-148	6.25	1.5625	0.25		
Pazufloxacin	6.25	1.5625	0.25	0.75	Partial Synergy
SAAP-148	6.25	3.125	0.5		
Prulifloxacin	3.125	1.5625	0.5	1	Additivity
SAAP-148	6.25	3.125	0.5		
Sisomicin	0.78125	0.3906	0.5	1	Additivity
SAAP-148	6.25	3.125	0.5		
Aztreonam	6.25	3.125	0.5	1	Additivity
SAAP-148	6.25	3.125	0.5		
Sparfloxacin	6.25	3.125	0.5	1.5	Indifference
SAAP-148	6.25	6.25	1		
Pefloxacin	12.5	6.25	0.5	1	Additivity
SAAP-148	6.25	3.125	0.5		
Besifloxacin	12.5	6.25	0.5	1	Additivity
SAAP-148	6.25	3.125	0.5		
Azithromycin	50	25	0.5	1	Additivity
SAAP-148	6.25	3.125	0.5		
Chlorhexidine	12.5	6.25	0.5	1	Additivity
SAAP-148	6.25	3.125	0.5		
Netilmicin	3.125	1.5625	0.5	1	Additivity
SAAP-148	6.25	3.125	0.5		
Piperacillin	1.5625	0.78125	0.5	1	Additivity
SAAP-148	6.25	3.125	0.5		
Cefozopran	0.78125	0.78125	1	1.5	Indifference
SAAP-148	6.25	3.125	0.5		
Sitafloxacin	1.5625	1.5625	1	2	Indifference
SAAP-148	6.25	6.25	1		
Amikacin	1.5625	0.78125	0.5	1.5	Indifference
SAAP-148	6.25	6.25	1		
Gemifloxacin	3.125	1.5625	0.5	1	Additivity
SAAP-148	6.25	3.25	0.5		
Cefmenoxime	3.125	1.5625	0.5	1	Additivity
SAAP-148	6.25	3.125	0.5		
Lomefloxacin	6.25	3.125	0.5	1	Additivity
SAAP-148	6.25	3.125	0.5		
Garenoxacin	6.25	3.125	0.5	1	Additivity
SAAP-148	6.25	3.125	0.5		
Gatifloxacin	6.25	3.125	0.5	1	Additivity
SAAP-148	6.25	3.125	0.5		
Levofloxacin	12.5	6.25	0.5	1.5	Indifference
SAAP-148	6.25	6.25	1		
Enoxacin	12.5	6.25	0.5	1.5	Indifference
SAAP-148	6.25	6.25	1		
Danofloxacin	6.25	3.125	0.5	1.5	Indifference
SAAP-148	6.25	6.25	1		
Tosufloxacin	3.125	1.5625	0.5	1.5	Indifference
SAAP-148	6.25	6.25	1		
Streptomycin	3.125	1.5625	0.5	1.5	Indifference
SAAP-148	6.25	6.25	1		
Doripenem	1.5625	1.5625	1	2	Indifference
SAAP-148	6.25	6.25	1		
Colistin	1.5625	0.78125	0.5	1.5	Indifference
SAAP-148	6.25	6.25	1		
Ceftazidime	1.5625	1.5625	1	2	Indifference
SAAP-148	6.25	6.25	1		
Rifaximin	12.5	6.25	0.5	1.5	Indifference
SAAP-148	6.25	6.25	1		
Erythromycin	50	50	1	2	Indifference
SAAP-148	6.25	6.25	1		
Telithromycin	50	25	0.5	1.5	Indifference
SAAP-148	6.25	6.25	1		
Bekanamycin	12.5	6.25	0.5	1.5	Indifference
SAAP-148	6.25	6.25	1		
Tobramycin	0.78125	0.78125	1	2	Indifference
SAAP-148	6.25	6.25	1		
Paromomycin	12.5	12.5	1	2	Indifference
SAAP-148	6.25	6.25	1		

*FICI is the fractional inhibitory concentration index.*

### Time-Kill Curve Assay for the *P. aeruginosa*

**FIGURE 3:**
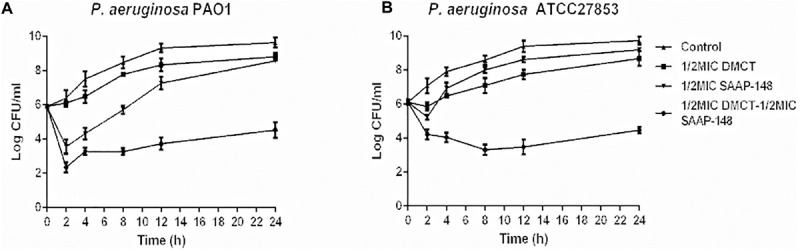
Time-kill curves for DMCT and SAAP-148 alone or combination against *P. aeruginosa*. Time-kill assay using one-second the MIC of each agent (6.25 μM of DMCT, 3.125 μM of SAAP-148) alone and in combination for PAO1 **(A)** and ATCC27853 **(B)**. The data were presented as mean ± SD.

The time-kill assays were performed in order to confirm the synergistic effects of SAAP-148 and DMCT against PAO1 and ATCC27853. As shown in Figure 3, the SAAP-148 (1/2 × MIC) and DMCT (1/2 × MIC) alone did not induce cell death after 24 h incubation. The combination of DMCT(1/2 × MIC) and SAAP-148 (1/2 × MIC) showed a reduced viable count (> 3log10 CFU/ml) compared to that of the initial inoculum. Although the regrowth was observed at 24 h, the combined treatment was still at least ≥ 5log10 CFU/ml lower than the level of growth control at 24 h (Figure 3). Overall, combination therapy can significantly reduce the bacterial count compared with DMCT and SAAP-148 monotherapy.

### Therapeutic Efficacy of DMCT Combined With SAAP-148 *in vivo*

**FIGURE 4:**
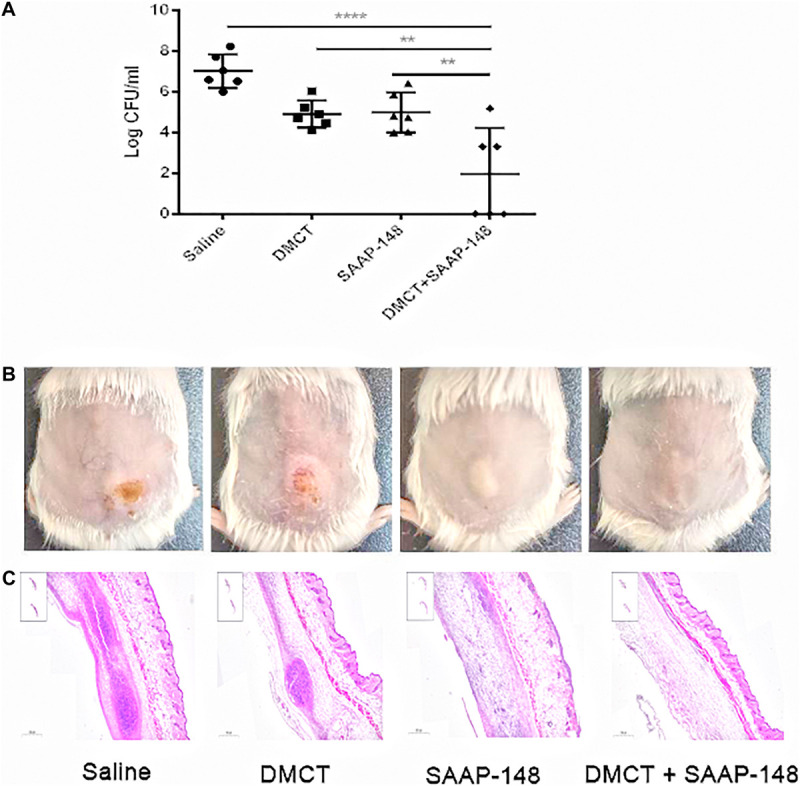
DMCT and SAAP-148 combinatorial therapy in a murine cutaneous abscess model. CD-1 female mice were injected with 1 × 10^9^ CFU/ml *P. aeruginosa* PAO1 and treated 1 h post-infection with saline (control), DMCT, SAAP-148, or DMCT + SAAP-148 combination. Drugs concentrations for all conditions were as follows: DMCT, 5 mg/kg and SAAP-148, 10 mg/kg. Infected abscess lumps and pus were excised to determine CFU by 3 days post-infection. Counted CFU/ml data is expressed **(A)**. An entire dorsal back abscess and representative results are shown at 3 days after therapy for PAO1 **(B)**. **(C)** Histological results (H&E stain, 3×, down panels) are shown at 3 days after therapy for mono- or combined therapy. All experiments were performed three times with 2–4 mice per group. Statistical analysis was performed using one-way ANOVA, Kruskal–Wallis test with Dunn’s correction (two-sided). The asterisk indicates significant differences. **P < 0.01; ****P < 0.0001.

Finally, we used murine model of cutaneous abscesses to determine the *in vivo* efficacy of the combination therapy. Infections with high bacterial loads (> 10^7^ CFU/ml bacteria) are rarely mentioned, especially those associated with biofilms or abscesses, but deserve more attention (Pletzer et al., 2018). The effects of different drugs concentrations were determined through preliminary experiments, with reference to the pharmacokinetics or empirically test (Agwuh and Macgowan, 2006; de Breij et al., 2018). We finally determined appropriate concentration that reduces abscess areas enough to observe the synergistic effect between SAAP-148 and DMCT (Supplementary Figure S2). The combination of DMCT and SAAP-148 against PAO1 were about 36 times lower than the saline control group and showed synergistic effects over the monotherapy (Figure 4). Similarly, DMCT (5 mg/kg) or SAAP-148 (10 mg/kg) could not inhibit the growth of abscess alone, but abscess size was notably diminished when used in combination. Consistent with these results, histopathological analysis showed that compared with monotherapy, the group of mice treated with the combination therapy significantly reduced the inflammation of the skin abscess, or even disappeared.

These important observations prove that antimicrobial monotherapy is usually ineffective when *P. aeruginosa* develops high-density infections. In addition, combination therapy can significantly enhance the efficacy of bacterial infections.

## Discussion

The purpose of this work was to find possible inhibitors against *P. aeruginosa*, which is notorious for its increased resistance. The thirty-nine drugs were selected through high-throughput screening, and their antibacterial effects on *P. aeruginosa* were further evaluated through phenotypic experiments. In addition, we showed that the combination of antimicrobial peptide SAAP-148 and DMCT showed a strong antimicrobial effect on both the standard strain of *P. aeruginosa* and the clinically isolated strain of MDR.

High-throughput screening has become an important approach in finding target drugs. Moreover, the plans of repurposing existing drugs that are currently being used as treatments for other diseases is also attractive (Miró-Canturri et al., 2019). Successful repurposing screens have produced candidates for Zika virus and Ebola virus (Johansen et al., 2015; Barrows et al., 2016). In this study, we screened these drugs like many other studies in order to find drugs that can inhibit *P. aeruginosa*. It has been listed by the World Health Organization as an important pathogen that poses a “serious” threat to human health (Shankar, 2014). The ability to form intrinsic resistant biofilms is posing another major threat to human health, as several infections include catheter-related infections and wounds directly related to biofilms. Therefore, we focused on finding drugs that have inhibitory effects on biofilms. Among the selected drugs, 37 drugs inhibited biofilm formation by more than 80% at the concentration of 30 μM. We further screened out 18 of 39 drugs that disrupt the preformed biofilms (Figure 1). The destruction of biofilm is the key to the treatment of chronic infection of *P. aeruginosa*. These drugs may play an important role in the treatment of biofilm diseases.

We found that the combination of DMCT and SAAP-148 has a synergistic effect on *P. aeruginosa*. SAAP-148 is derived from the classical AMP LL-37 with improved antimicrobial and antibiofilm activities. Compared with many antimicrobial peptides in the preclinical and clinical stages, SAAP-148 has higher *in vitro* bactericidal efficacy under physiological conditions. SAAP-148 showed broad antibacterial activity against both Gram-positive and Gram-negative bacteria of MDR and could interact with cell membrane and permeate rapidly. In particular, SAAP-148 can kill persister cells and eradicate biofilm without selective resistance. DMCT is a tetracycline derivative antibiotic produced by Lederle et al. (McCormick et al., 1957). DMCT kill bacteria by binding to ribosomes inhibiting protein synthesis. It is used as a broad-spectrum antibiotic, but may be more often used to treat chronic syndrome of inappropriate secretion of antidiuretic hormone (SIADH) (Miell et al., 2015). According to reports, DMCT is expected to be used as a contrast agent for intraoperative detection of brain tumors (Sarkar et al., 2020). In our study, we collected 6 MDR clinical isolate strains of *P. aeruginosa* for combination therapy. It is worth noting that we found that the combination of DMCT and SAAP-148 has a synergistic effect on these strains (Supplementary Table S3). It is known that the killing of bacteria by SAAP-148 involves the rapid interaction of peptides with the bacterial membrane and subsequent permeabilization, leading to the death of bacteria. Thus, we infer that the mode of action of SAAP-148 likely facilitates the penetration of DMCT to the bacteria, and at the next step, the interaction of DMCT and SAAP-148 causes death of bacteria.

The bacterial skin and soft tissue infections (SSTIs) are a very common problem. The bacteria that often cause SSTI are methicillin-resistant Staphylococcus aureus (MRSA), but gram-negative bacteria are becoming more and more important in global SSTI (Ruef, 2008; Mansour et al., 2016). The SENTRY Antimicrobial Surveillance Program (North America) reported that 10.8% of the major pathogens isolated from SSTI include *P. aeruginosa* (Rennie et al., 2003). SSTIs can lead to cutaneous abscesses, and recurring abscesses cause difficult-to-treat infections. According to our research, highly synergistic interactions between DMCT and SAAP-148 were observed against a high bacterial load-containing abscess model of PAO1. In our cutaneous abscess model, DMCT or SAAP-148 alone showed a moderate effect on bacterial load. The abscess area of the monotherapy group was similar to that of the saline control group (Figure 4). On the contrary, DMCT combined with SAAP-148 could inhibit abscess and reduce PAO1 bacterial load. There is a good correlation between bacterial burden reduction and combination therapy. Topical application of the peptide SAAP-148 in hypromellose gel at a dose up to 300 mg/day per animal, proved to be safe without any adverse effects (de Breij et al., 2018). As an ancient antibacterial drug, the safety of DMCT *in vivo* animal research and clinical application has been fully proved, though DMCT is more widely used in non-antibacterial fields. In the screening trial, subjects were given DMCT 600 mg/day by oral therapy for pustules treatment (Plewig and Schöpf, 1975). The combination of antibiotics and antimicrobial peptides targeting bacterial membrane can effectively treat infection and reduce the severity of skin abscess.

In summary, we used high-throughput screening methods to identify drugs that have bactericidal activity against *P. aeruginosa*. A total of 39 compounds were identified as potential drugs against PAO1, which were further confirmed by phenotypic experiments. It was also concluded that the combination therapy resulted in a significant reduction in the effective concentrations of DMCT and SAAP-148 needed to inhibit *P. aeruginosa* and reduce its toxicity. Our results proved that DMCT in combination with SAAP-148 proved to be an attractive option for the development of antimicrobial combined therapy.

## Data Availability Statement

The original contributions presented in the study are included in the article/Supplementary Material, further inquiries can be directed to the corresponding author.

## Ethics Statement

The animal study was reviewed and approved by the Clinical samples collection and animal experiments were conducted under the approval of the Ethics Committee of the Third Xiangya Hospital, Central South University (No. 2019-S021). Strains were isolated from clinical samples routinely collected from patients, and the identification of patients was not needed. Therefore, the need for written informed consent was waived and oral informed consent was obtained.

## Author Contributions

YW, PS, and SL conducted the experimental design for this study. SL was the main completer of the experiment, analyzed the results of the experiment, and wrote the manuscript. LC provided reagents and methods needed for the study. LZ, XZ, LX, and YL performed some supplementary experiments. YW supervised the entire study. All the authors read and approved the final manuscript.

## Conflict of Interest

The authors declare that the research was conducted in the absence of any commercial or financial relationships that could be construed as a potential conflict of interest.

## Supplementary Material

Supplementary Figure 1 Hemolytic activity of SAAP-148. 5% (v/v) hRBCs were treated with serially diluted SAAP-148 for 1 h at 37°C, then the absorbance of 100 μl supernatants was measured at 570 nm to calculate the hemolysis activity. 1% DMSO with 1 × PBS was a negative control, and 0.1% Triton X-100 was a positive control. ^****^*P* < 0.0001.

Supplementary Figure 2 Murine cutaneous abscess model by using monotherapy. Mice were infected with 1 × 10^9^ CFU PAO1 to form abscess infection. And then treated intra-abscess with either no drugs (saline control), DMCT (1, 5,10, 20 mg/kg) **(A)** or SAAP-148 (1, 5, 10, 20 mg/kg) **(B)** after 1 h post-infection, the abscess tissue was measured by colony count after 3 days post-infection. All experiments were conducted three times with 2–4 mice per group.
