# Supplementary material for: High-Throughput Identification of Antibacterials Against Pseudomonas aeruginosa
Source: Front Microbiol. 2020 Dec 9;11:591426. doi: 10.3389/fmicb.2020.591426 (PMC7755642; doi:10.3389/fmicb.2020.591426)
Supplement: Supplementary Figure 1 — Hemolytic activity of SAAP-148. 5% (v/v) hRBCs were treated with serially diluted SAAP-148 for 1 h at 37°C, then the absorbance of 100 μl supernatants was measured at 570 nm to calculate the hemolysis activity. 1% DMSO with 1 × PBS was a negative control, and 0.1% Triton X-100 was a positive control. ****P < 0.0001. [file Data_Sheet_1.docx]

Supplementary Material

# Supplementary Figures and Tables

## Supplementary Figures





**Supplementary Figure S1. Hemolytic activity of SAAP-148.** 5% (v/v) hRBCs were treated with serially diluted SAAP-148 for 1 h at 37°C, then the absorbance of 100µl supernatants was measured at 570 nm to calculate the hemolysis activity. 1% DMSO with 1 × PBS was a negative control, and 0.1% Triton X-100 was a positive control. ****, *P*<0.0001.


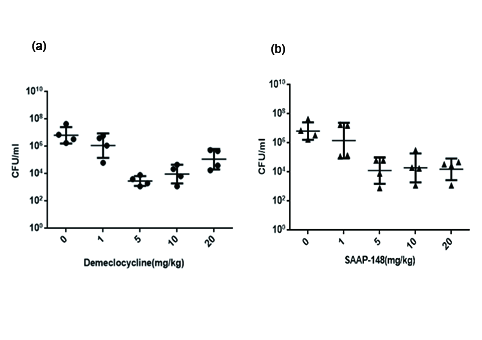


**Supplementary Figure S2.** **Murine cutaneous abscess model by using monotherapy.** Mice were subcutaneously infected with 1×10^9^ CFU *P. aeruginosa* PAO1. And then treated intra-abscess with either no drugs (saline control), DMCT (1, 5,10, 20 mg/kg) (a) or SAAP-148 (1, 5, 10, 20 mg/kg) (b) after 1 h post-infection, the abscess tissue was measured by colony count after 3 days post-infection. All experiments were performed three times with 2-4 mice/group.

## Supplementary Tables

**Supplementary Table S1. Results of the second high-throughput screening.**

The detailes of the second screening are displayed in Excel form.

**Supplementary Table S2. MIC of antimicrobial peptides against PAO1.**

| Drug | MIC (µM) |
| --- | --- |
| SAAP-148 | 6.25 |
| SAAP-159 | 6.25 |
| SAAP-149 | 12.5 |
| SAAP-276 | 12.5 |
| GH12 | 12.5 |
| LL-37 | >50 |

**Supplementary table S3. FICs of DMCT and SAAP-148 alone or in combination against *P. aeruginosa* clinical isolation strain.**

| Clinical isolation strain | MIC (µM) | | FICI | Outcome |
| --- | --- | --- | --- | --- |
|  | DMCT | SAAP-148 |  |  |
| PA017 | 12.5 | 12.5 | 0.75 | Additive |
| PA018 | 12.5 | 12.5 | 0.75 | Additive |
| PA029 | 6.25 | 12.5 | 0.75 | Additive |
| PA035 | 25 | 12.5 | 0.75 | Additive |
| PA044 | 12.5 | 12.5 | 0.75 | Additive |
| PA046 | 12.5 | 12.5 | 0.75 | Additive |
